# A Comparison of Different Textured and Non-Textured Anti-Reflective Coatings for Planar Monolithic Silicon-Perovskite Tandem Solar Cells

**DOI:** 10.1021/acsaem.2c00361

**Published:** 2022-05-12

**Authors:** Michael Spence, Richard Hammond, Adam Pockett, Zhengfei Wei, Andrew Johnson, Trystan Watson, Matthew J. Carnie

**Affiliations:** † Department of Materials Science & Engineering and SPECIFIC-IKC, Swansea University, Bay Campus, Fabian Way, Swansea SA1 8EN, UK; ‡ IQE Silicon Compounds, Beech House, Pascal Close, St. Mellons, Cardiff CF3 0LW, UK; § IQE Europe Ltd., Pascal Close, St. Mellons, Cardiff CF3 0LW, UK

**Keywords:** ARC, PDMS, perovskite, silicon, tandem

## Abstract

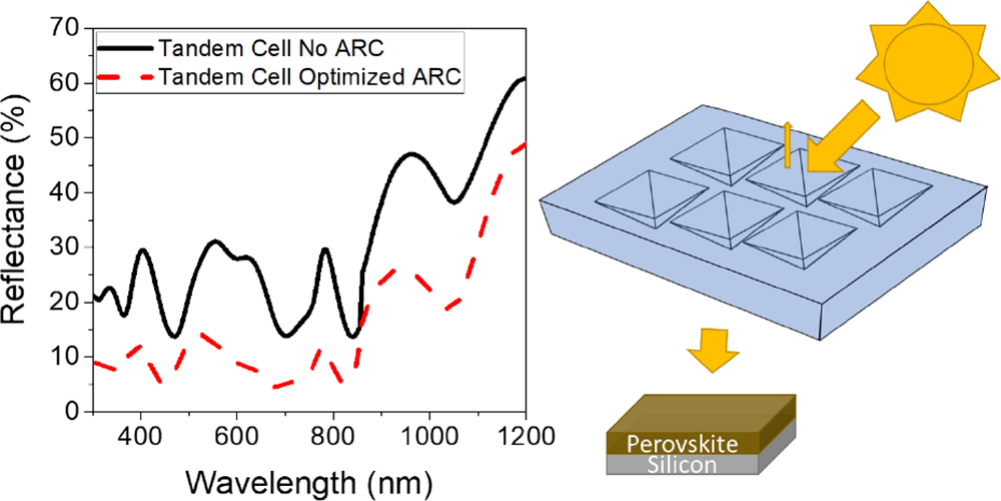

Multijunction solar cells offer a route to exceed the Shockley–Queisser limit for single-junction devices. In a few short years, silicon-perovskite tandems have significantly passed the efficiency of the best silicon single-junction cells. For scalable solution processing of silicon-perovskite tandem devices, with the avoidance of vacuum processing steps, a flat silicon sub-cell is normally required. This results in a flat top surface that can lead to higher optical reflection losses than conformal deposition on textured silicon bottom cells. To overcome this, textured anti-reflective coatings (ARCs) can be used on top of the finished cell, with textured polydimethylsiloxane (PDMS), a promising candidate. In this work, we vary the texture geometry and film thickness of PDMS anti-reflective foils to understand the effect of these parameters on reflectance of the foil. The best film is selected, and anti-reflective performance is compared with two common planar ARCs—lithium fluoride (LiF) and magnesium fluoride (MgF_2_) showing considerable reduction in reflectance for a non-textured silicon-perovskite tandem cell. The application of a PDMS film is shown to give a 3–5% increase in integrated *J*_SC_ in each sub-cell of a silicon-perovskite tandem structure.

## Introduction

Silicon-perovskite tandems combine up and coming perovskite thin film materials with established and widespread silicon technologies, with silicon-perovskite tandems reaching a record 29.5% in 2020.^1^ Silicon perovskite tandems can be divided into two types: parallel four-terminal devices that consist of a perovskite cell mechanically stacked on a silicon device and connected separately and two-terminal devices where the perovskite device is fabricated directly on a silicon cell. Two-terminal monolithic devices possess the advantages over four-terminal devices of simpler processing and reduced parasitic absorption due to requiring fewer interlayers;^2^ however, they must be carefully designed to ensure current matching in both sub-cells. Many of these devices have been produced by conformal deposition, usually vacuum deposition,^3^ on a silicon sub-cell with a textured top surface to reduce reflection losses; however, this excludes standard solution processing of the cell such as spin coating, slot die coating, or blade coating as these cannot produce conformal layers. Solution processing of the perovskite on smaller silicon cell texturization features can be achieved, but this results in thick perovskite layers,^4^ which results in a flat top surface with poorer anti-reflective performance.^5^ The silicon sub-cell with a planar top allows standard solution processing, which facilitates the highest efficiency perovskite films in single-junction devices^6,7^ and allows for low-cost, scalable manufacturing of perovskite devices.^8^ Many of the most efficient devices on flat silicon bottom cells have used anti-reflective coatings (ARCs) to mitigate some of these reflection losses.^3^ Simulations by Altazin and colleagues^5^ of a planar n-i-p silicon-perovskite tandem on untextured silicon show a significant improvement in generated currents of ∼8% in the perovskite sub-cell and ∼16% in the silicon sub-cell by addition of an anti-reflective coating.

Commercially common planar films such as LiF and MgF_2_ have been used to improve the performance of tandem cells^9−11^ due to their favorable refractive indices.^12,13^ Though planar anti-reflective layers act to improve the performance of flat tandem cells, the improvement in photocurrent is less than achieved with both silicon texturing and an anti-reflective coating.^14^ This is because surface texturization acts to increase the proportion of reflected light that bounces back onto the surface rather than away from the substrate, increasing the chance that it will be absorbed and thus providing a complimentary mechanism for anti-reflectance. Increasingly, textured anti-reflective films have been turned to as a potential solution to provide this light trapping on flat surfaces and have been shown to be effective in silicon-perovskite tandem cells.^15,16^

A range of patterns from biologically inspired nanofur^17^ and rose petal texture^18^ to patterns that replicate the upright pyramid structures of textured silicon cells^19^ have been used on such films to improve cell performance. Of these, random pyramid textures are possibly the simplest type of structures to produce as the molds are based on relatively simple selective etching techniques, which are already widely employed in silicon PV manufacture. While a relatively accessible technique, upright pyramid structures first require the imprinting of inverted molds or stamps from the textured silicon. Once cured, these molds or stamps are then used to apply the random upright pyramid pattern to the anti-reflective foil. An even simpler technique, as shown by Kuo and colleagues,^20^ is to spin-coat a curable material directly onto the textured silicon wafer, which results in an anti-reflective foil with an inverted pyramid texturization. In addition to reducing the number of process steps, random inverted pyramid textures applied to photovoltaic cells have been shown to outperform upright pyramids.^21^

In this paper, we study the optical properties of textured and non-textured anti-reflective films. Polydimethylsiloxane (PDMS) was selected to produce the anti-reflective foils for this work. PDMS is used in a number of studies to produce textured anti-reflective films^15,18,20,22^ and has numerous advantages, such as being fast curing, low cost, commercially available, and not requiring vacuum deposition. We prepared planar and inverted pyramid textured PDMS foils and investigated the effect of film thickness on the optical performance of each type of foil. The size distribution of the inverted pyramid sizes is reported by Hou et al.^22^ to have an impact on anti-reflective properties, and so, we also produce PDMS foils with two different average pyramid sizes. Both thickness and pyramid size are shown to impact on the anti-reflective properties of the anti-reflective coating and must be considered to optimize the foil. The best performing textured anti-reflective layer was applied to a non-textured silicon-perovskite tandem and overall reflection compared with common planar anti-reflective layers. Quantum efficiency measurements of the same tandem show clear improvements in integrated photocurrent after the application of the textured anti-reflective foil.

## Experimental Section

### Planar PDMS Films

Planar PDMS films were produced on 1.1 mm-thick soda-lime glass substrates. Substrates were cleaned by sonication in a mixture of 2% Hellmanex and deionized water for 15 min followed by rinsing sequentially in acetone and isopropyl alcohol before drying with nitrogen. A two-part PDMS kit (DOWSIL Sylgard 184) was mixed in a weight ratio of 10:1 base to curing agent and degassed using a vacuum desiccator. The PDMS mixture was deposited in the center of the glass substrate and spin-coated at speeds of between 500 and 6000 rpm for 30 s to achieve the range of layer thicknesses. The films were cured for 1 h on a hotplate at 100 °C as it was found that a manufacture-specified curing time of 35 min was not sufficient for the thickest films.

### Textured PDMS Films

Textured PDMS films were fabricated using textured silicon molds. To fabricate the molds, ⟨100⟩ silicon was etched in a 10% weight:weight solution of sodium hydroxide (Sigma-Aldrich, Reagent Grade 97% flakes) in a 4:1 volume:volume mixture of deionized water and isopropyl alcohol at 70 °C. The etch time was varied from 15 to 60 min to produce the micron scale and sub-micron scale molds. To allow easy removal of the textured PDMS films, the molds were silinized by spray coating with trimethoxy(octyl)silane (TMOS) (Sigma-Aldrich, 99%) and dried on a hotplate at 60 °C for 30 min prior to spin coating. Without this pre-treatment, it was found to be very difficult to remove large-area 30 mm × 30 mm films without damage. The textured films were prepared in the same manner as the planar films with a spin speed of 1000 rpm used. After annealing, the films were detached by slowly peeling from one corner to the other using tweezers. The films were then turned over so that the inverted pyramid texturization faced upward and laminated onto either a glass substrate (UV–vis measurements) or directly onto the photovoltaic cell.

### Fabrication of Tandem Cells

A non-textured silicon bottom cell was used to allow for solution processing of the tandem cell. Polished silicon homojunction wafers were provided by IQE plc and cleaved to produce individual substrates of 25 mm × 25 mm. To form the recombination layer, an indium zinc oxide layer of 30 nm was deposited on top of this cell using a Moorfield MiniLab 60 sputtering system. The silicon sub-cells were then sonicated in a Hellmanex mixture before rinsing in acetone and isopropyl alcohol as described for the glass substrates. Immediately prior to deposition of the first layer of the perovskite sub-cell, the silicon sub-cells were treated with an O_2_ plasma for 10 min to aid wetting.

To form the electron transport layer, SnO_2_ nanoparticles (Alfa Aesar, 15% in H_2_O) were diluted in deionized water in a ratio of 1:3 and were spin-coated onto the substrate at 3000 rpm for 30 s before annealing on a hotplate at 140 °C for 45 min in air. The cells were transferred to a nitrogen-filled glovebox for deposition of the perovskite and hole transport layer. A 1.25 M solution of CH_3_NH_3_I (Greatcell Solar) and PbI_2_ (TCI Chemicals) with a 5% PbI_2_ excess was prepared in a mixture of 4:1 (v:v) DMF:DMSO. A precursor solution of 100 μL was deposited onto the substrate and spin-coated at 4000 rpm for 30 s. After 6 s, 200 μL of ethyl acetate was dropped onto the spinning substrate to aid crystallization of the perovskite. The films were then annealed on a hotplate for 10 min at 100 °C. To produce the hole transport layer, a 90 mg mL^–1^ solution of Spiro-OMeTAD (Sigma-Aldrich) was prepared in chlorobenzene. To improve the conductivity of the film, the solution was doped by adding 34 μL mL^–1^ t-BP, 19 μL mL^–1^ LiTFSI (1.8 M in acetonitrile), and 10 μL mL^–1^ FK 209 Co(III) TFSI salt (0.25 M in acetonitrile). The precursor (50 μL) was dynamically deposited onto a substrate spinning at 4000 rpm and left to spin for an additional 10 s.

To protect the hole transport layer from sputter damage, devices were transferred to a MBraun PROvap thermal evaporator where a 10 nm layer of MoO_*x*_ (Kurt J. Lesker, 99.95% purity) was evaporated on top of the hole transport layer at a rate of 0.15 Å s^–1^. A 200 nm layer of indium zinc oxide was then sputter-coated through a shadow mask to form a transparent top contact. Finally, the cells were transferred to an Edwards E306 bell jar evaporator where silver (Kurt J. Lesker, 99.99% pellets) was evaporated onto the rear of the cell to form a back contact. Perovskite single-junction devices were fabricated on ITO-coated glass following the same process but omitting the MoO_*x*_ and IZO deposition stages.

For the LiF and MgF_2_ anti-reflective coated devices, 100 nm LiF (Kurt J. Lesker, 99.5%, powder) or MgF_2_ (Kurt J. Lesker, 99.9 9%, pieces) was evaporated on top of the device stack in a MBraun PROvap thermal evaporator at a deposition rate of 1.0 Å s^–1^.

### Characterization

Quantum efficiency measurements were carried out using a PV Measurements QEX10 solar cell quantum efficiency measurement system in AC mode. The light intensity of the lamp was determined prior to measurement using calibrated silicon and germanium reference diodes. For characterization of the tandem device, the response of each sub-cell was measured separately. To measure the silicon sub-cell, the device was illuminated by a blue LED (450 nm) during the AC quantum efficiency measurement to ensure sufficient current in the perovskite sub-cell, as to not limit the current response of the silicon sub-cell. To measure the perovskite sub-cell, an infrared LED (850 nm) was used in the same way to bias the silicon sub-cell during measurements. Additionally, for the perovskite measurements, the cell was forward-biased to compensate for the low shunt resistance of the perovskite sub-cell and provide short circuit conditions during the measurement.^9^

Transmittance and reflectance spectra were obtained using a PerkinElmer Lambda 750 UV–vis–NIR spectrophotometer with an integrating sphere attachment calibrated prior to measurement using a certified Spectralon 99% diffuse reflectance standard. Film thicknesses were measured using a KLA Tencor D-600 profilometer. SEM images of textured films and molds were taken using a Hitachi TM3000 desktop SEM.

## Results and Discussion

**Figure 1:**
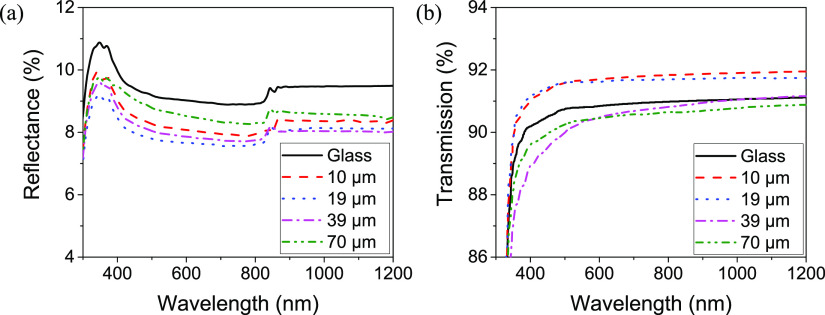
(a) Percentage reflectance and (b) percentage transmission spectra for planar PDMS layers of different thicknesses coated on soda-lime glass substrates.

To understand the optical properties of the PDMS material, planar PDMS films were produced on glass by spin coating, as described in the Experimental Section, to enable transmission and reflectance measurements. Different spin speeds were used to produce films of different thicknesses, and the measured film thickness vs spin speed is given in Table 1. UV–vis–NIR transmission and reflectance measurements were taken for each film and are given in Figure 1. To confirm the repeatability of the measurement system, multiple transmittance and reflectance spectra were taken for a glass control sample over the course of an hour, showing a standard deviation of less than 0.01 T% and 0.01 R% in mean transmittance and reflectance over the measured wavelength range.

**Table 1:** PDMS Film Thicknesses for Different Spin Speeds, Measured by a Profilometer

spin speed	500 rpm	1000 rpm	3000 rpm	6000 rpm
film thickness	70.1 μm	39.1 μm	18.9 μm	9.9 μm

The film thicknesses ranged from approximately 10 to 70 μm. To understand the reflectance and transmittance performance in the context of photovoltaic performance, we used an average weighted reflectance to the AM1.5G solar spectrum following the method of Kuo and colleagues.^20^ Solar weighted average reflectance and transmittance are given in eqs 1 and 2, respectively:
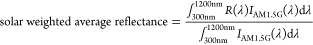
1
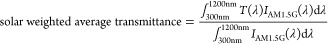
2

All films provided reduced reflectance over a bare glass substrate with no ARC, with the minimum reflectance achieved with the 19 μm-thick planar layer, which had a solar weighted average reflectance value of 89% of the bare glass value. Transmittance measurements showed improved transmission over the glass substrate alone for the 10 and 19 μm films; however, a slight reduction in transmission was seen for the glass coated with the two thicker films. This can be attributed to the increase in parasitic absorption as the film thickness increases, which begins to outweigh the reduced reflectance at greater thicknesses. This effect is highlighted for the 10 and 19 μm films, where despite the reflectance of the 19 μm film being the lower of the two, increased parasitic absorption causes its percentage transmission to be slightly poorer than the thinner 10 μm film (Table 2).

**Table 2:** Mean Transmittance and Reflectance for Each Thickness of PDMS over a Wavelength Range of 300–1200 nm

	solar weighted average R%	solar weighted average T%
glass	9.29	90.31
10 μm	8.28	91.17
19 μm	7.92	91.08
39 μm	8.03	89.94
70 μm	8.62	89.95

**Figure 2:**
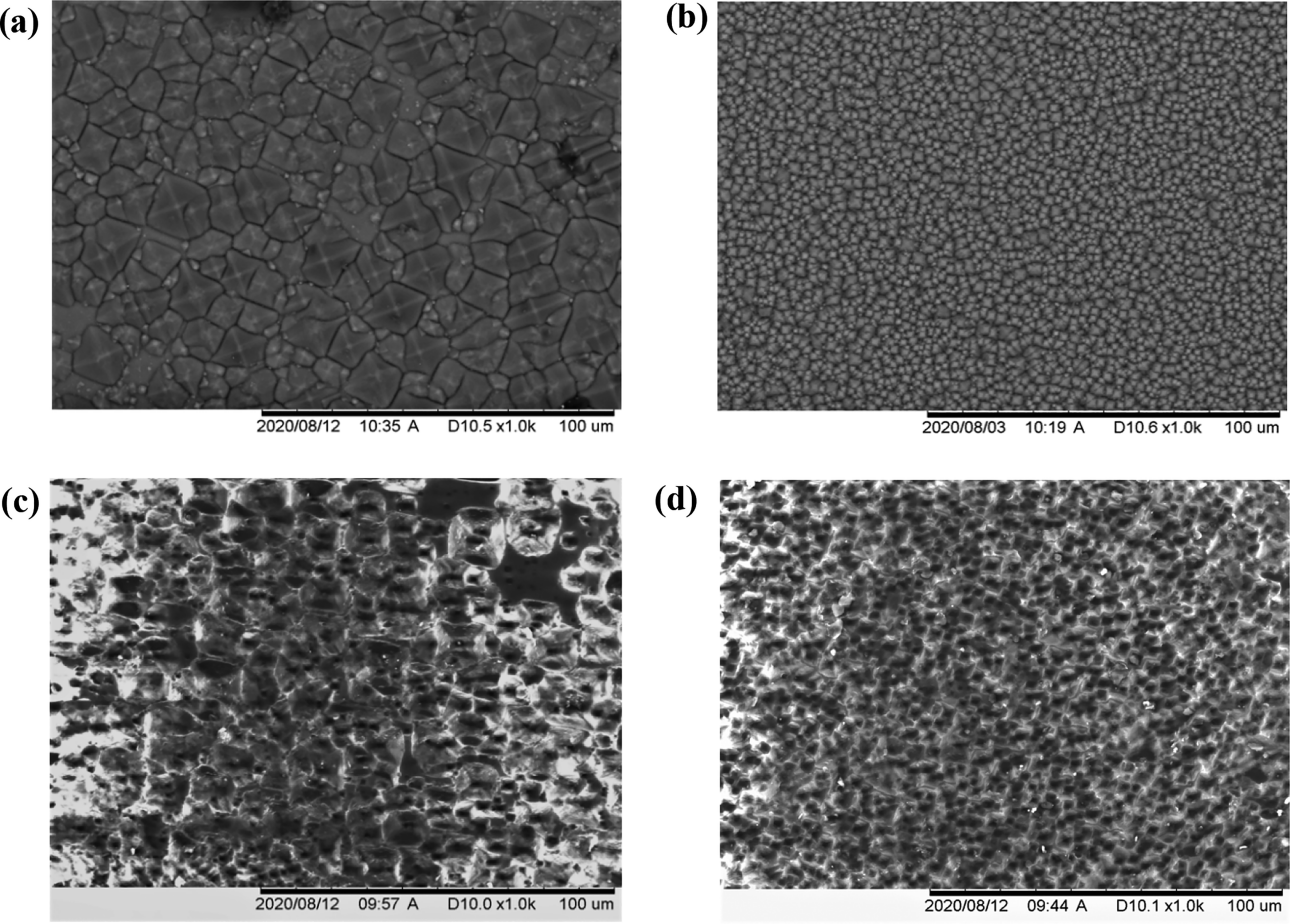
Scanning electron micrographs of (a) micron (T1) and (b) sub-micron (T2) scale silicon molds and the corresponding (c) micron and (d) sub-micron scale inverted pyramid textured PDMS layers produced by these molds.

To produce the textured films, silicon molds were prepared as described in the Experimental Section. Silicon molds with micron scale (T1) and sub-micron scale (T2) random pyramid textures were created by varying the KOH etch time. The distribution of pyramid heights was obtained by measuring 20 pyramids at different locations across the sample using SEM images and ImageJ imaging software and calculating the height trigonometrically. For mold T1, the median pyramid height ranged from 7 to 10 μm, while for mold T2, the median pyramid height was in the 500 nm to 1 μm range with a mix of nano- and microscale pyramids. Distribution histograms of measured pyramid heights are given in the Supporting Information in Figure S1. Figure 2 shows example SEMs of molds T1 and T2 and the resulting PDMS foils produced with them. It can be seen from this figure that, for both molds, many of the pyramids overlap, resulting in overlapping inverted pyramids on the foils. It has previously been shown by Chen et al.^23^ by ray tracing simulations that the exact way in which the inverted pyramids overlap also affects their anti-reflective properties. Thus, by varying just one parameter, etching time, two significantly different anti-reflective textures can be produced.

A spin speed of 3000 rpm was chosen for the textured anti-reflective films as this provided the highest reflectance of all the measured planar films and close to the highest transmission, resulting in a film thicknesses of approximately 19 μm for both substrates. Second, this spin speed produced foils that were considerably easier to peel and handle than those produced with the fastest speed. Figure 8a shows an intact, textured 19 μm PDMS film of approximately 30 mm × 30 mm, which has been laminated on a glass substrate. As these films are textured, it is important to note that the reported thickness values are average film thicknesses^24^ taking into account the depths of the inverted pyramids, and hence, the thickness at any given point can vary by up to plus or minus half the maximum pyramid depth.

**Figure 3:**
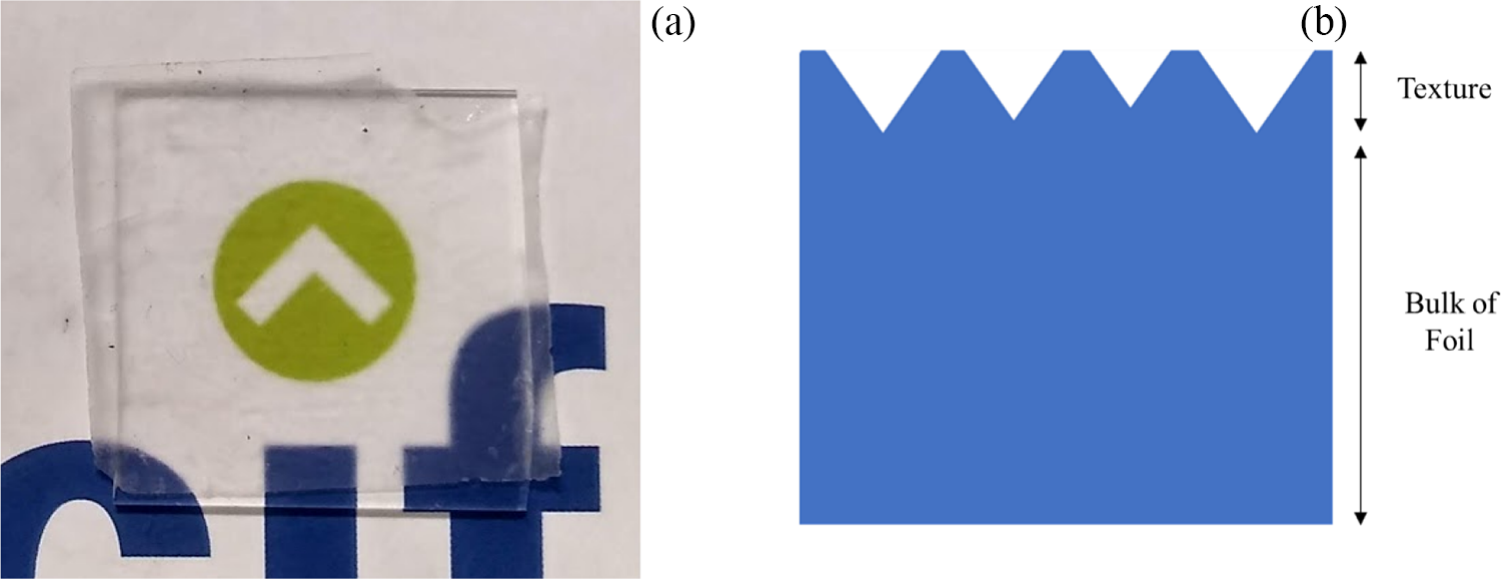
(a) Fully delaminated textured PDMS film of approximately 30 mm × 30 mm showing a haze effect due to the scattering of transmitted light. (b) Schematic of the foil showing the inverted random pyramid texturization on a thicker bulk dependent on coating speed.

**Figure 4:**
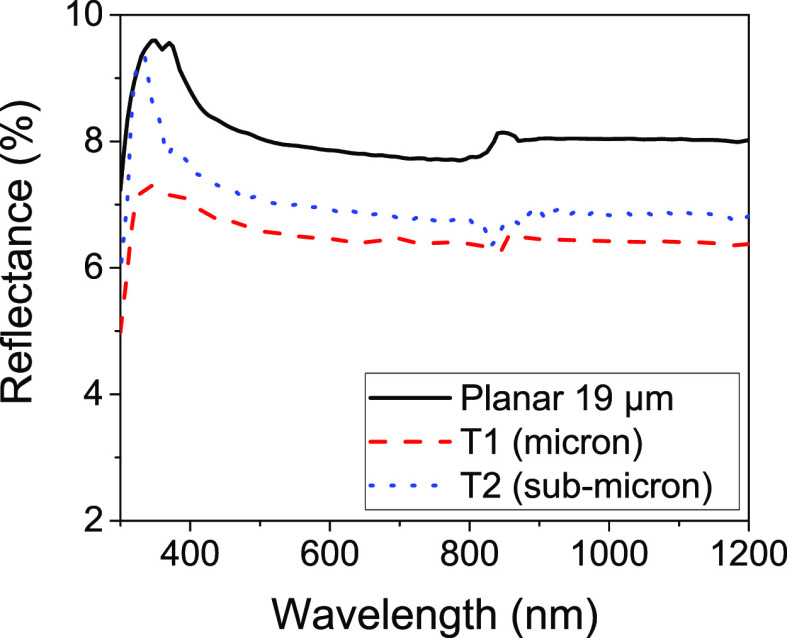
Reflectance spectra of micron and sub-micron textured anti-reflective films coated at 3000 rpm compared to a planar film coated under the same conditions giving films of similar thickness.

The two textured PDMS anti-reflective foils T1 and T2 were flipped and laminated onto soda-lime glass so that the textured side faced upward, as shown in Figure 3b, and reflectance measurements were taken as before. Figure 4 compares the reflectance of the two different textured films T1 and T2 with a planar film of the same thickness.

The textured films show much improved anti-reflective properties over the planar film resulting in weighted average reflectances of 70 and 74%, respectively, for T1 and T2 compared to the bare glass substrate, as can be seen in Figure 4 and Table 3.

**Table 3:** Mean Reflectance for Each Thickness of PDMS for Wavelengths of 300–1200 nm

	mean R%
texture 1	6.49
texture 2	6.92
planar	7.92

The larger-scale inverted pyramid structure of T1 gives improved transmittance across the measured spectrum over the sub-micron textured film T2. The difference between the two texturizations is most noticeable in the 300–350 nm wavelength range where T1 shows a significant reduction in reflectance compared to the planar film, while the smaller-scale texturized film shows a much more modest improvement. This is in good agreement with the work of Wu et al. who compared different size distributions of inverted pyramid structures etched onto the surface of mono-crystalline silicon cells and found an improvement in reflectance as the average pyramid sizes increase from the sub-micron to micron scale.^21^

**Figure 5:**
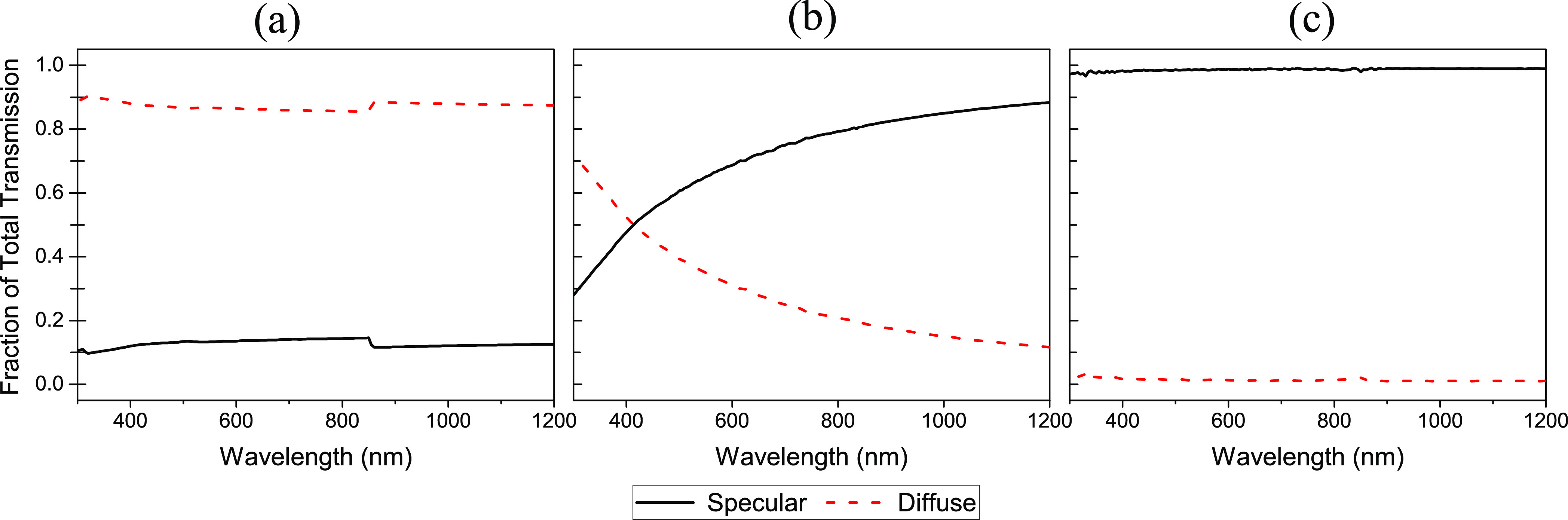
Diffuse and specular components of transmission for the (a) T1 textured film, (b) T2 textured film, and (c) planar film as a fraction of total transmission. Films were all produced with the same spin speed of 3000 rpm for comparison giving films approximately 19 μm thick.

By measuring the transmittance of the films, we gain some insights into how the textured anti-reflective layers interact with incoming light. Specular and total reflectances were measured following the method described by Yan et al.,^17^ and diffuse reflectance was calculated by subtracting the specular component from the total. These results are presented in Figure 5. For the planar film, the specular component makes up almost all (99%) of the transmitted light; however, for the textured film T1, the trend is reversed, and the transmitted light is mostly diffused with the specular component only around 13% of the total. The film with the smaller texture T2 shows an intermediate case with specular light making up 72% of the total transmission, with the fraction of diffuse and specular reflectances showing a large variation across the spectrum.

The random nature of the pyramid textured surface acts to scatter the incoming light in many directions,^25^ diffusing the transmitted light. A portion of light reflected off the surface undergoes multiple reflections before being transmitted, further contributing to the diffuse nature of the transmitted light.^26^ For the planar film, due to the collimated nature of the incident light from the spectrometer and the smooth surface of the film, most of the light will either be directly transmitted or undergo specular reflection. In the larger texture (T1), a much higher fraction of the transmitted light is diffused than for the smaller texture (T2). This suggests that a greater proportion of the light undergoes multiple reflections, which could explain the superior anti-reflective performance of T1. In T2, a greater proportion of pyramids are of a similar size to the wavelengths of the measured spectrum, where the geometric optics approximation is no longer valid, and diffractive effects become important. This can degrade anti-reflective performance compared to larger inverted pyramids as discussed by Han and colleagues^27^ and also reduce the proportion of light that undergoes multiples reflections, resulting in the lower diffuse transmittance.

**Figure 6:**
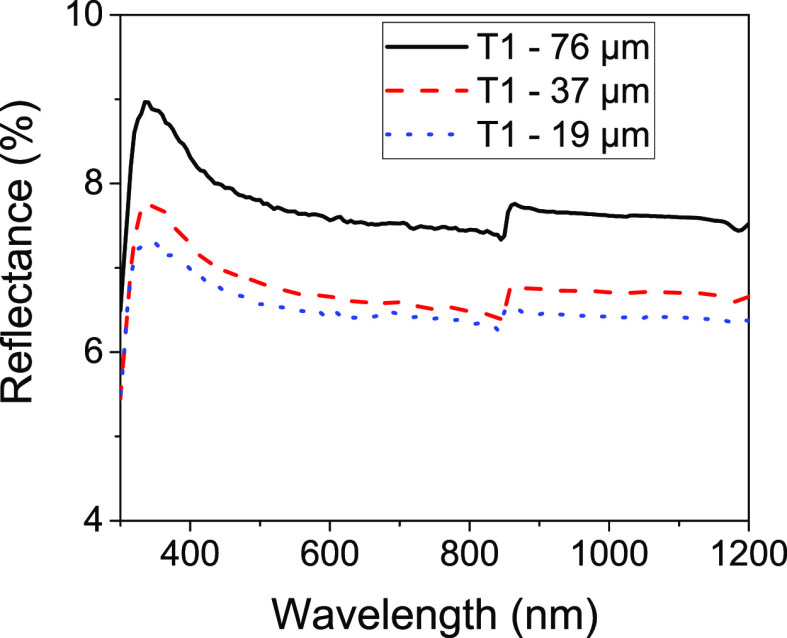
Reflectance spectra for T1 micron scale textured PDMS films of different thicknesses. Films (6000 rpm) were not measured due to the difficulty in delaminating such thin foils intact from the substrate.

The spin speed selected for the processing of the textured films was 3000 rpm based on the process conditions for the planar film with the lowest reflectance. This makes the assumption that the reflectance of the textured foils follows the same trend as the planar films and decreases with increasing spin speed (lower thickness) up to 3000 rpm (∼19 μm). To explore whether this assumption holds, foils with the better performing texture (T1) were produced by spin coating at different spin speeds, as with the planar films and reflectance spectra measured (Figure 6). Foils were produced at 500, 1000, and 3000 rpm resulting in average film thicknesses of 76, 37, and 19 μm, respectively. When coating at 6000 rpm, it was not possible to delaminate a sufficiently large section of the foil for testing due to its very low thickness.

Reflectance spectra of the textured films show a similar trend of improvement with decreasing thickness with the 19 μm film producing the lowest reflectance of the films tested. Compared with the 19 μm film, there is a significant increase in reflectance of 18% for a 76 μm film (500 rpm) and a much smaller increase of 4% with a 37 μm (1000 rpm) film. Development of better delamination and handling techniques for thinner films could show whether reducing the thickness of the film further improves the anti-reflective properties; however, as the improvement between 37 and 19 μm is already relatively modest, any improvement from increasing spin speed needs to be balanced with the decreased robustness of the thinner foil.

**Figure 7:**
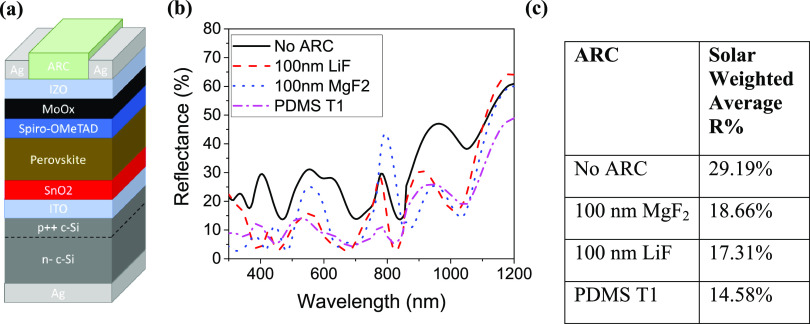
(a) Schematic of the flat surface silicon-perovskite tandem used for evaluation of different anti-reflective coatings. (b) Reflectance spectra of the flat surface silicon-perovskite tandem with the optimized micron scale PDMS ARC (T1) compared with the commonly used planar ARC. (c) Tabulated mean reflectance over 300–1200 nm for the silicon-perovskite tandem with each ARC.

To evaluate the performance of the textured PDMS anti-reflective layer, n-i-p silicon-perovskite tandem cells were fabricated on a non-textured silicon sub-cell. Figure 7a shows a schematic of the flat surfaced tandem cell consisting of a top cell with solution-processed perovskite and charge transport layers and a silicon homojunction cell fabricated from a polished silicon wafer to allow the aforementioned solution processing.

Due to its superior anti-reflective properties, the larger textured PDMS anti-reflective layer T1 was chosen as the most suitable PDMS ARC to apply to the non-textured tandem cell. LiF and MgF_2_ are commonly used to produce planar anti-reflective coatings and have previously been used on high efficiency silicon-perovskite tandem cells to further improve performance. Further, tandem cells were produced with 100 nm LiF and 100 nm MgF_2_ applied as anti-reflective coatings to compare with the textured ARC.

Figure 7b shows the reflectance spectra of the silicon-perovskite tandems with (i) no anti-reflective coating, (ii) MgF_2_ planar ARC, (iii) LiF planar ARC, and (iv) the T1 micron scale textured PDMS anti-reflective layer. It can be seen from Figure 7 that, despite the presence of the fairly rough perovskite top cell, the tandem structure has high reflectance across the 300–1200 nm range, with an average weighted reflectance of 29.19%. The tandem device with the textured PDMS anti-reflective layer shows the lowest solar weighted average reflectance at 14.58%, with the LiF and MgF_2_ showing improved weighted average reflectances of 17.31 and 18.66%, respectively, but still poorer than the textured PDMS ARC.

The planar anti-reflective layers do show some significant improvements in reflectance over the device with no ARC but show less broadband reduction in reflectance than the PDMS layer. The LiF shows little reduction in reflectance at wavelengths above 1100 nm and below 350 nm with a reflectance peak at 770 nm. The MgF_2_ has a slightly higher average reflectance than the LiF and also suffers from large reflectance peaks around 550 and 800 nm as well as little reduction in reflectance above 1100 nm. The textured PDMS ARC reduces reflectance of the cell across the 300–1200 nm range. This is an important feature for monolithic tandems where the current in both sub-cells must be closely matched as they are connected in series. By achieving a strong reduction in reflection (>20%) across almost the entire spectrum at which the device absorbs, the textured PDMS ARC should provide an improvement to generated *J*_SC_ in both sub-cells, which in turn means that an improvement in the total *J*_SC_ is possible without further optimization of the cell.

**Figure 8:**
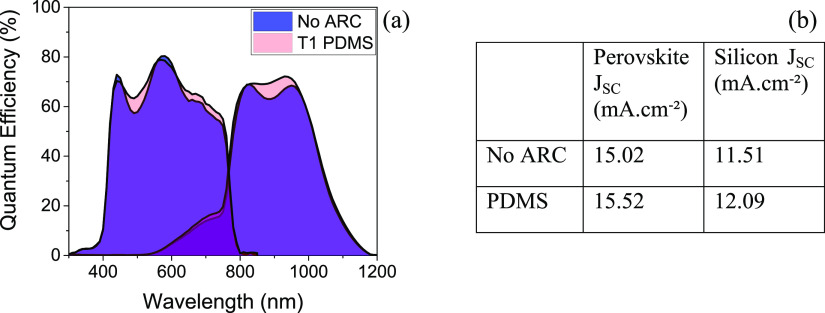
(a) Quantum efficiency plot of a flat top silicon-perovskite tandem cell before (shaded blue) and after (shaded pink) lamination of the best performing textured silicon ARC. The area shaded in purple shows the overlap of the two traces. (b) Integrated *J*_SC_ for each sub-cell with and without the textured PDMS ARC.

External quantum efficiency (EQE) measurements were taken to understand the effect of the textured PDMS anti-reflective foil on cell performance. As perovskite cells in particular are prone to variability in performance from device to device,^28^ the same cell was used for both measurements, and JV curves for this device are given in the Supporting Information, Figure S2. The quantum efficiency spectrum was measured for the device with no ARC layer before the textured T1 PDMS ARC was carefully laminated onto the cell, and the measurement was repeated. The quantum efficiency spectra are given in Figure 8a. For both sub-cells, application of the textured PDMS layer shows an improvement in efficiency across most of the spectral range.

The *J*_SC_ values of the perovskite and silicon sub-cells with and without the ARC were calculated by integration of the quantum efficiency spectra and are shown in Figure 8b. It is worth noting that this silicon-perovskite tandem has not been optimized for current matching, and consequently, the perovskite *J*_SC_ is significantly higher than the silicon *J*_SC_ for both cases. The silicon shows a larger improvement of 0.58 mA cm^–2^ or 5% with application of the textured PDMS anti-reflective coating due to a small but significant increase in EQE between 850 and 1000 nm. The perovskite shows a slightly smaller absolute increase of 0.50 mA cm^–2^, which results in a smaller percentage increase in an integrated *J*_SC_ of 3% due to its higher initial *J*_SC_. The improvement in total generated current density (the sum of the perovskite and silicon currents) of 1.08 mA/cm^–2^ after application of the textured PDMS ARC to the tandem is slightly larger than achieved by Bush and colleagues^15^ and much higher in relative terms but is lower than some others such as Park et al.^29^ who use a PDMS nanofur. Most of the improvement in quantum efficiency in the perovskite sub-cell, which contributes to this *J*_SC_, comes in the 450–520 and 650–750 nm ranges with slight reductions in quantum efficiency in some parts of the spectrum. These reductions in quantum efficiency are likely attributable to parasitic absorption in the PDMS layer as the reflectance of the device with the textured PDMS ARC is much lower than the no-ARC device at these wavelengths. Little improvement is seen at ultraviolet wavelengths as the quantum efficiency is extremely low due to the strong parasitic absorption of the spiro-OMeTAD layer in this range;^30^ however, for devices with a p-i-n structure or n-i-p devices with a more transmissive hole transport layer, an improvement in quantum efficiency might be expected with the PDMS anti-reflective layer, resulting in further enhancement of *J*_SC_ for these device structures. To understand this further, we produced a >19% efficient small area perovskite single-junction cell on ITO glass following the same method as for the tandem cell. As the spiro-OMeTAD and MoO_*x*_ layers are below the bottom of the cell and the highly transparent thin SnO_2_ film is at the top, parasitic absorption is significantly improved. Quantum efficiency measurements of this device show an improvement in integrated *J*_SC_ of +1.14 mA cm^2^ or 6% relative improvement, bringing it in line with the result for the silicon sub-cell in the tandem.

## Conclusions

We have presented a simple method for the fabrication of an inverted pyramid textured PDMS film, which is compatible with silicon-perovskite tandem solar cells. Various thicknesses of the planar film were compared, finding that optimal reflectance losses were achieved with a 19 μm film produced by spin coating at 3000 rpm. A larger texture geometry with an average pyramid height of 8 μm was found to have lower reflection losses than a film mainly composed of sub-micron pyramids. In terms of total film thickness, the thinnest foil, which could be delaminated, resulted in the best anti-reflective properties. The textured film produced with the best thickness and texture height shows favorable anti-reflective performance compared to two commonly used inorganic planar ARCs, lithium fluoride and magnesium fluoride when applied to a silicon-perovskite tandem. Application of the best performing textured PDMS ARC produced almost 50% reduction in average reflectance for a silicon-perovskite tandem with a planar top surface. This resulted in a modest but significant increase in an integrated *J*_SC_ of >0.5 mA cm^–2^ or 3 and 5% relative increases, respectively, in both perovskite and silicon sub-cells for the measured tandem device. Minimization of parasitic absorbances in the tandem cell is likely to amplify this improvement further, with a 6% relative increase in integrated *J*_SC_ by applying the film to an optimized single-junction perovskite cell. In conclusion, both the geometry of the texture and total film thickness impact the anti-reflective performance of the PDMS layer. With some optimization, a textured PDMS film gives improved performance to common planar anti-reflective coatings and helps mitigate the reflection losses in non-textured silicon-perovskite tandem cells, allowing solution processing.

## Supporting Information Available

Pyramid texture size distributions, JV curves, and additional QE data (PDF)

## Notes

The data that support these findings are openly available in the Swansea University data archive at https://doi.org/10.5281/zenodo.5948624.

## ABBREVIATIONS

ARC: anti-reflective coating
PDMS: polydimethylsiloxane
DMF: *N*,*N*-dimethylformamide
DMSO: dimethyl sulfoxide
Li-TFSI: lithium bis(trifluoromethylsulfonyl)imide
FK 209 Co(III) TFSI salt: tris(2-(1*H*-pyrazol-1-yl)-4-*tert*-butylpyridine)cobalt(III) tri[bis(trifluoromethane)sulfonimide]
IZO: indium zinc oxide
