# Supplementary material for: A Comparison of Different Textured and Non-Textured Anti-Reflective Coatings for Planar Monolithic Silicon-Perovskite Tandem Solar Cells
Source: ACS Appl Energy Mater. 2022 May 12;5(5):5974–82. doi: 10.1021/acsaem.2c00361 (PMC9131309; doi:10.1021/acsaem.2c00361)
Supplement: Supplementary file 1 — ae2c00361_si_001.pdf [file ae2c00361_si_001.pdf]

Supporting Information

*Michael Spence, Richard Hammond, Adam Pockett, Zhengfei Wei, Andrew Johnson, Trystan Watson and Matthew J. Carnie**

SPECIFIC, Swansea University Bay Campus, Swansea SA1 8EN, UK

ASSOCIATED CONTENT

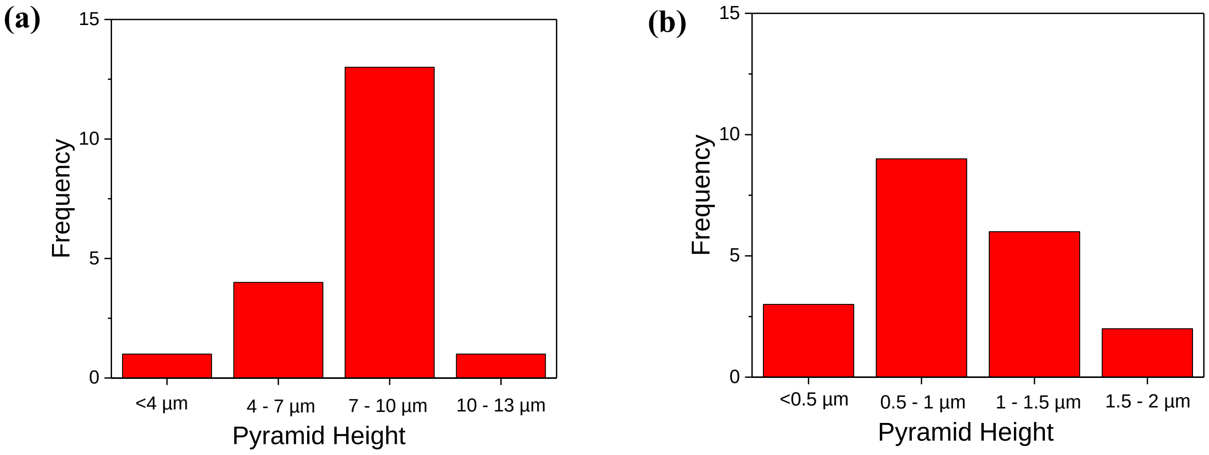

Figure S1. Histograms of pyramid height distributions for silicon mold (a) T1 and (b) T2

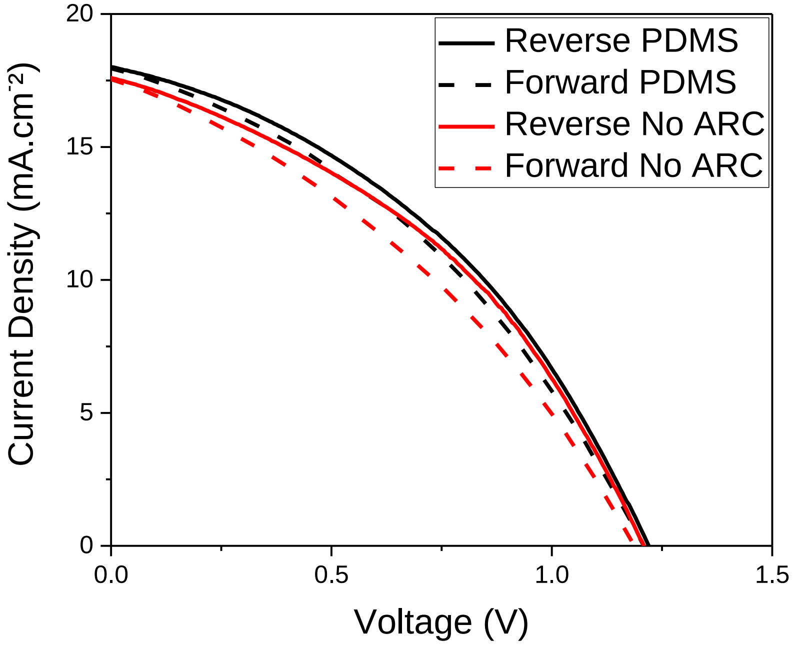

Figure S2. Current density vs. voltage curves for an NIP tandem cell before and after application of the textured PDMS films.

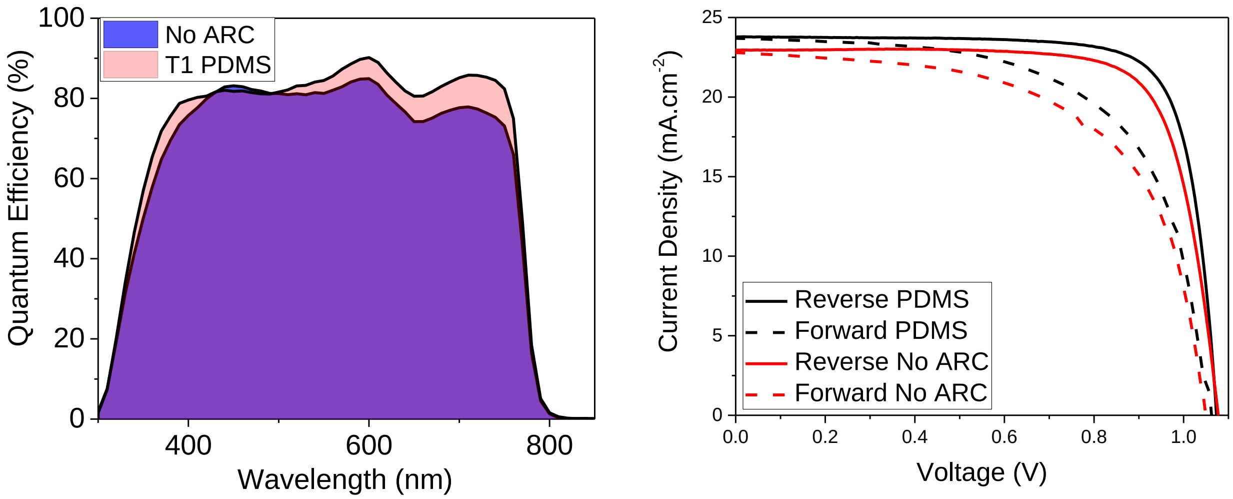

(a) **(b)**

Figure S3. (a) Quantum efficiency and (b) current density vs. voltage curves for an NIP single junction cell before and after application of the textured PDMS films.
